# Supplementary material for: Information transfer in mammalian glycan-based communication
Source: eLife. 2023 Feb 20;12:e69415. doi: 10.7554/eLife.69415 (PMC10014076; doi:10.7554/eLife.69415)
Supplement: Supplementary file 1. [file elife-69415-supp1.docx]

Supplementary File 1: List of antibodies

| Antigen | Supplier | Product Number |
| --- | --- | --- |
| dectin-2 | biotechene | FAB3114P |
| MINCLE | Sho Yamasaki | N/A |
| DC-SIGN | BioLegend | 330105 |
| MCL | Miltenyi Biotec | 60522010 |
| TNFAR | Miltenyi Biotec | 130-120-149 |
| TLR1 | ThermoFisher | 12-9011-80 |
| TLR2 | Miltenyi Biotec | 130-099-017 |
| dectin-1 | BioLegend | 355403 |
| MGL | Miltenyi Biotec | 130-109-641 |
| Anti-FcεRI Antibody, γ subunit-FITC | Millipore Sigma | FCABS400F |
