# Supplementary material for: Information transfer in mammalian glycan-based communication
Source: eLife. 2023 Feb 20;12:e69415. doi: 10.7554/eLife.69415 (PMC10014076; doi:10.7554/eLife.69415)
Supplement: Supplementary file 2. [file elife-69415-supp2.docx]

Supplementary File 2: List of primers

| GOI |  | Sequence 5'-3' |  |  |
| --- | --- | --- | --- | --- |
|  | **pLV-EF1a-IRES-Hygro/Bla/Neo** |  |  |  |
| DC-SIGN | PFw pLV DC-SIGN | gtcgtgaggatccACCACCatgagtgactccaag | |  |
|  | PRv pLV DC-SIGN | cctcgaggaattctcactacgcaggaggggggt | |  |
| FcRγ | FcRγ fw | gtcgtgaggatccACCACCatgattccagcagt | |  |
|  | FcRγ rv | cctcgagGAATTCctactgtggtggtttc | |  |
| MCL | Pfw MCL | gtcgtgaggatccACCACCatggggctagaaaaa | |  |
|  | Prv MCL | cctcgagGAATTCctagttcaatgttgttcca | |  |
| dectin-1 | Pfw d-1 | gtcgtgaggatccACCACCatggaatatcatcctgatttag | | |
|  | Prv d-1 | cctcgagGAATTCttacattgaaaacttcttctcac | |  |
|  | **RP172** |  |  |  |
| mincle | MINCLE Fw | GAGCTAGCAGTATTAATTAACCACCatgaattcatctaaatcatc | | |
|  | MINCLE Rv | GTACCGGTTAGGATGCATGCTCAttaaagagattttcctttg | | |
| dectin-2 | dectin-2 Fw | GAGCTAGCAGTATTAATTAACCACCatgatgcaagagcagcaac | | |
|  | dectin-2 Rv | GTACCGGTTAGGATGCATGCTCAataggtaaatcttattcatc | | |
